# Comparison of six-week flywheel and traditional resistance training on deceleration and dynamic balance in elite badminton players

**DOI:** 10.3389/fphys.2025.1491661

**Published:** 2025-02-25

**Authors:** Shurui Yuan, Zepeng Lu, Shiwen Tan, Zijie Zhang, Shiwei Jing, Haoyang Liu, Zhihui Zhou, Dapeng Bao

**Affiliations:** ^1^ Sports Coaching College, Beijing Sport University, Beijing, China; ^2^ China Football College, Beijing Sport University, Beijing, China; ^3^ School of Sports Engineering, Beijing Sport University, Beijing, China; ^4^ China Institute of Sport and Health Science, Beijing Sport University, Beijing, China

**Keywords:** flywheel resistance training, deceleration deficit, dynamic posture stability, badminton athletes, strength training

## Abstract

**Introduction:**

The aim of this study was to compare the effects of flywheel resistance training (FRT) and traditional resistance training (TRT) on deceleration and dynamic balance performance in elite badminton players.

**Methods:**

Seventeen elite male badminton players (age: 21.36 ± 2.10 years) volunteered to participate and were randomly assigned to either a flywheel resistance training group (FT: n = 8) or a traditional resistance training group (RT: n = 9). The FT group performed flywheel resistance training twice a week for 6 weeks, while the RT group participated in traditional resistance training for the same period. Deceleration and dynamic balance performance were assessed at baseline and following the intervention using the dynamic posture stability index (DPSI) test, 5-0-5 change of direction (COD) test, deceleration deficit (DD) test, and isometric mid-thigh pull (IMTP) test.

**Results and Discussion:**

Repeated-measures ANOVA revealed a significant time × group interaction for DPSI of both legs and peak force in the IMTP test (*p* < 0.05, partial *η* ^2^ = 0.42–0.79), with better post-test performance compared to pre-test result in the FT group (ES = 0.30–2.10), and the improvements were higher than that of the RT group. No significant differences were observed in the DD test and COD test between FT and RT groups (*p* > 0.05); however, the magnitude of improvement in DD for the FT group (ES = 0.99) was greater than that of the RT group (ES = 0.52). This pilot study demonstrates that, compared to traditional resistance training, flywheel resistance training enhances deceleration performance and improves dynamic balance in elite badminton players.

## Introduction

Badminton is a racket sport characterized by short-duration, high-intensity actions and brief rest periods within a match (Cabello Manrique and González-Badillo, 2003; Phomsoupha and Laffaye, 2015). Studies have found that athletes change direction 8–10 times per minute during matches, which demands quick COD, horizontal accelerations and decelerations, jumps, and lunges at the net from various postural positions (Hong et al., 2014; Phomsoupha and Laffaye, 2015; Shariff et al., 2009). Deceleration and dynamic balance control are fundamental for executing rapid COD maneuvers, and these abilities are crucial for controlling the center of gravity, and ensuring movement continuity, thereby enhancing technical and tactical performance (Dos'Santos et al., 2021; DosʼSantos et al., 2017; Lu et al., 2022; Zhou et al., 2022). Moreover, common lower limb injuries in badminton are closely associated with deceleration and dynamic balance control (Phomsoupha and Laffaye, 2020). Research indicates that anterior cruciate ligament (ACL) injuries frequently occur during early deceleration before changing direction, rapid deceleration while sprinting, and near-straight-leg landings (Boden et al., 2000). Intense horizontal decelerations (<−3 m·s^−2^) strongly contribute to muscle damage in random intermittent multi-directional sports (de Hoyo et al., 2016; Gastin et al., 2019; Russell et al., 2016; Young et al., 2012) and may heighten the risk of injury if not appropriately managed (Bowen et al., 2020; Harper et al., 2022; Jaspers et al., 2018; Osgnach et al., 2010). Therefore, strategies aimed at improving deceleration and dynamic balance hold great promise for enhancing the performance of badminton athletes and reducing the risk of injuries.

One effective strategy is TRT, which helps athletes enhance strength (Khazaei et al., 2023; Kibele and Behm, 2009), balance (Khazaei et al., 2023; Kibele and Behm, 2009; Wang et al., 2023), COD ability (Silva et al., 2015), and functional performance (Kovac et al., 2022). Studies have demonstrated that, on average, a 15% increase in 1RM results in a 1.3% improvement in COD abilities after 5–6 weeks of TRT (Silva et al., 2015). During the braking phase of directional changes, the eccentric strength of muscles such as the hip extensors, quadriceps, gastrocnemius, and hamstrings plays a crucial role (McCurdy et al., 2014; Smith et al., 2009). Eccentric muscle strength can enhance rapid horizontal deceleration ability, improve performance (Chaabene et al., 2018; Dos'Santos et al., 2018; Dos'Santos et al., 2021), and reduce injury risk (Donelon et al., 2020; Dos'Santos et al., 2019; Marques et al., 2020). However, TRT is limited by its inability to provide significant eccentric loads during the concentric phase, with research indicating that it can only achieve maximal eccentric loads of 40%–50% (Dudley et al., 1991). Given the fast pace and high frequency of COD in badminton, TRT may not fully address the specific demands of the sport.

In recent years, FRT has garnered significant attention as an innovative eccentric strength training method (Burgos-Jara et al., 2023). It has shown promising results in enhancing athletic performance, preventing sports injuries, and aiding in neurological and orthopedic rehabilitation (Monajati et al., 2021). FRT utilizes the principle of inertia to generate resistance, allowing for maximal development of eccentric muscle contraction (Norrbrand et al., 2008). This training method adapts dynamically to the athlete’s performance, generating adaptive resistance that can reach up to 125% during the eccentric phase and maintain this load consistently throughout each training set (Norrbrand et al., 2008), thereby addressing some limitations of TRT. Recent research supports the use of FRT in sports, indicating that it offers unique physiological responses, such as concentric and eccentric force (Coratella et al., 2019), COD performance (Beato et al., 2021a; Beato et al., 2021b; Maroto-Izquierdo et al., 2017b), balance control (Sanudo et al., 2019), and injury risk reduction (Askling et al., 2003; Beato and Dello Iacono, 2020; de Hoyo et al., 2015), compared to other resistance exercise modalities (Beato et al., 2021c). Additionally, Maroto-Izquierdo et al. (2017a) found that FRT significantly improved acceleration and deceleration abilities in professional handball players during sprints with changes of direction compared to TRT. However, contradictory findings exist; for example, Joey et al. (2020) reported that a 4-week FRT intervention did not significantly enhance basketball players’ COD abilities. Given these conflicting results, the efficacy of FRT in improving the deceleration and dynamic balance performance of badminton athletes warrants further investigation.

In this pilot randomized controlled study, we aimed to compare the effects of a 6-week FRT and TRT on deceleration ability and dynamic balance in elite badminton players. We hypothesized that the FRT protocol would induce greater improvements in deceleration ability and dynamic balance compared to traditional resistance training.

## Materials and methods

### Participants

**FIGURE 1:**
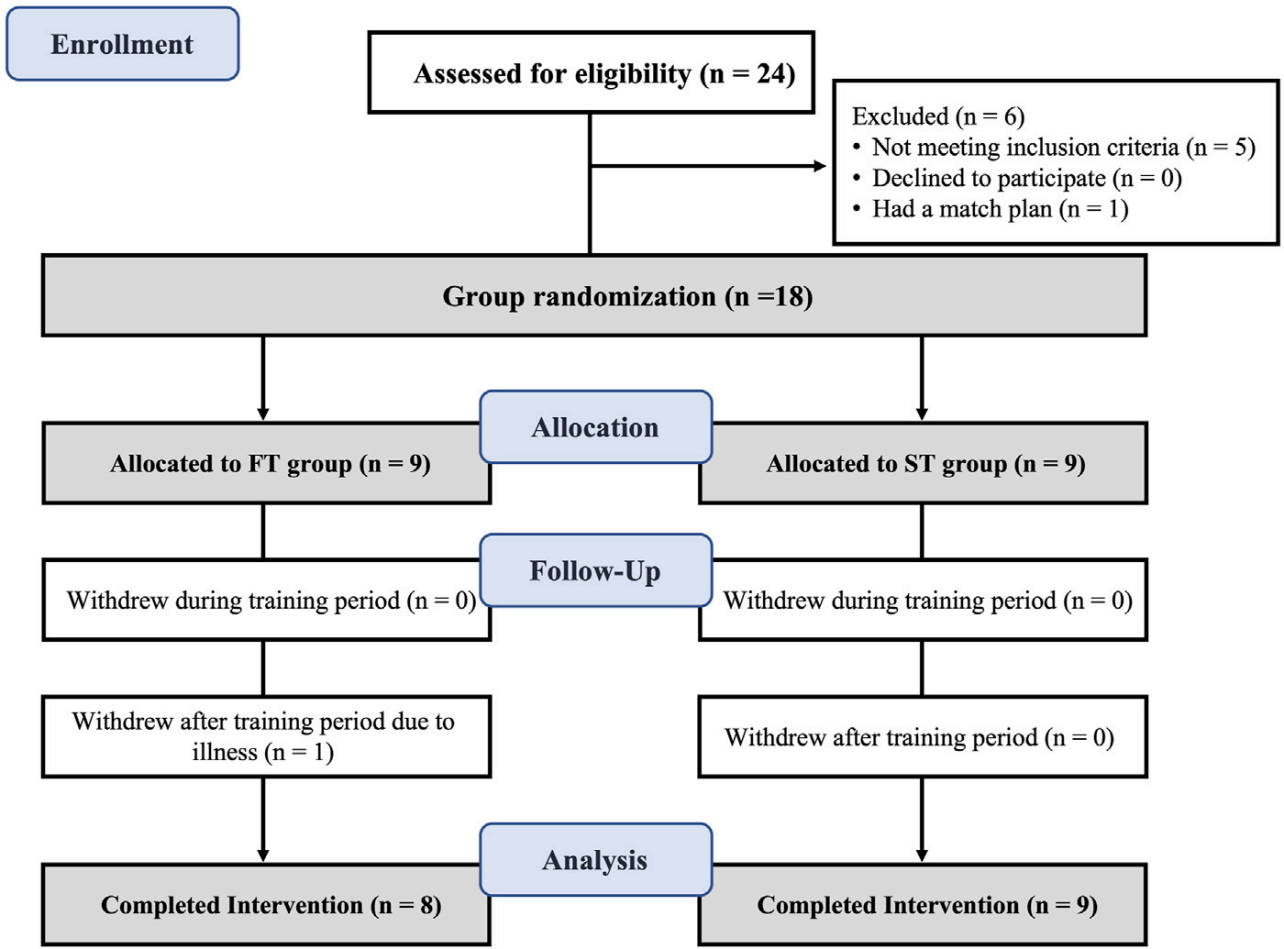
Flow diagram of the participants.

Statistical software G*Power 3.1.9.6 was used to determine the required sample size, setting the effect size (f) at 0.5 and the power at 0.95. The calculated sample size was no less than 16 participants. Ultimately, eighteen male badminton players were recruited for this study. The inclusion criteria were: (1) elite players who had played the quarterfinals of national youth games, the finals at the provincial games, or higher-level competitions; (2) a 1RM squat weight exceeding 1.5 times their body weight; and (3) the ability and willingness to complete the 6-week programs of test and training. The exclusion criteria were: participants who had ACL, hamstring, meniscus, ankle, or any other lower-extremity injuries in the 6 months prior to the experiment (See Figure 1 for participants recruitment process). The study protocol was approved by the Research Ethics Committee of Beijing Sport University (Approval number: 2023280H), and all procedures were conducted in accordance with the Declaration of Helsinki. Before data collection, the participants were informed of the benefits and potential risks related to the study, and all signed the informed consent form (Table 1).

**TABLE 1:** The demographic characteristics of the participants.

	Age (years)	Height (cm)	Weight (kg)	Training experience (years)
FT (n = 8)	21.57 ± 2.23	179.86 ± 6.04	75.00 ± 7.05	11.43 ± 1.81
RT (n = 9)	21.14 ± 2.12	180.43 ± 4.88	74.29 ± 5.40	11.86 ± 2.07

Note: Mean ± SD, was used for descriptive statistics.

### Study protocol

Eighteen players volunteered for random allocation to a flywheel resistance training group (FT, n = 9) or a traditional resistance training group (RT, n = 9). All participants in the FT and RT groups conducted the training program twice per week with 48 h of recovery between each session. Both groups underwent identical technical and tactical training, consisting of five sessions per week, each lasting approximately 2 h. The training focused specifically on skill development (e.g., footwork, stroke technique) and match simulations. Before the formal training and testing, all participants completed a 2-week familiarization period with flywheel exercises and resistance training exercises. Both groups performed the exercises in four sets of eight repetitions on either the flywheel machine (D Full, Desmotec, Italy) or using a free-weight barbell. After each set, participants were asked to self-report their effort on a modified scale of 1–10 (Hackett et al., 2012). The supervising trainers set the exercise load for both training groups was set to match a set-RPE of 8, which ensures that athletes achieve the training effect while maintaining acceptable performance. Specific method was to adjust the load on the barbell for the RT group or by encouraging participants to increase or decrease the speed of movement or adjust inertia (more/less flywheels) during FRT (Hackett et al., 2012). Details of the protocols of training were included in Table 2. Fully qualified trainers supervised all training sessions and every repetition performed by participants to ensure correct technical execution and exercise intensity.

**TABLE 2:** Training plans for FT and RT groups.

Training cycle	Group	Training content	Load plates	Load	Rest interval
Weeks 1–2	FT	Flywheel squat	P + L + M, L	4 sets (8 RPE)	3 min
		Flywheel deadlift	P, L		
	RT	Barbell squat	80% 1 RM	4 sets (8 RPE)	3 min
		Barbell deadlift			
Weeks 3–4	FT	Flywheel lunge	P + L + M, L	4 sets (8 RPE)	3 min
		Flywheel deadlift	P, L		
	RT	Barbell lunge	80% 1 RM	4 sets (8 RPE)	3 min
		Barbell deadlift			
Weeks 5–6	FT	Flywheel squat	P + L + M, L	4 sets (8 RPE)	3 min
		Flywheel lunge	P + L + M, L		
	RT	Barbell squat	80% 1 RM	4 sets (8 RPE)	3 min
		Barbell lunge			

Abbreviations: P, pro plate; L, large plate; M, medium plate; S, small plate.

### The assessment procedures

The tests were administered at baseline and within 3 days following the final session of the 6-week training program. To minimize the effects of fatigue, participants were instructed to refrain from high-intensity training for at least 24 h prior to testing. All assessments were conducted in laboratory setting, with a room temperature ranging from 20°C to 25°C and a relative humidity of 40%–50%. Researchers verbally explained the entire testing procedure and allowed players to familiarize themselves with the tests through 1-2 practice attempts. Players performed a Standardized warm-up routine to ensure they were in optimal condition and were then encouraged to exert maximum effort in each test. A 5–10-minute rest was given between each test. Each type of test was conducted at the same time and place across different visits, and the players were asked to wear the same sporting shoes they preferred throughout all the assessments.

### Deceleration deficit test

**FIGURE 2:**
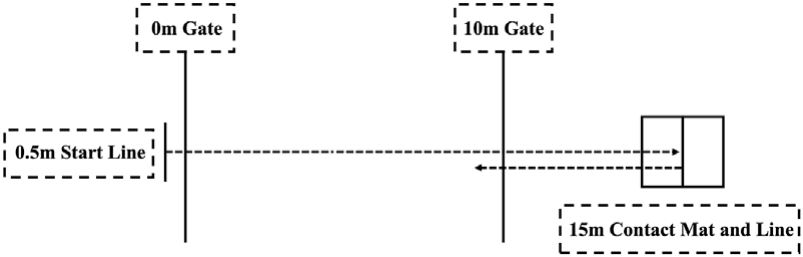
A Visual representation of the 5-0-5 COD test.

The DD was calculated to quantify the time an individual needed to come to a stop relative to their own sprinting speed (Clarke et al., 2022). As shown in Figure 2, two Smart Speed laser timers (Fusion Sports, Coopers Plains, Australia) are placed at the 0 m and 10 m lines. Additionally, a contact mat is placed at the 15 m finish line and secured to the floor, which is used to record commencement and duration of plant step ground contact. The full approach time was used in order to represent the time required to approach the turn line as fast as possible while decelerating into a position to facilitate exit speed and overall 5-0-5 performance (Clarke et al., 2022; Freitas et al., 2022). Players start from the same starting position, sprint to the 15 m finish line, and place their foot on the line marked at the center of the contact mat. They then perform a 180-degree turn and sprint back to the 10 m finish line. The full approach time and the ground contact time (GCT) are recorded.

15 m linear sprint time: Players start with their feet staggered, 0.5 m behind the first timing gate. After preparing, they sprint at full speed to the 15 m finish line, and the 15 m sprint time is recorded.

Each test is performed twice, and the shorter time is used for analysis. Players are given a 5-min rest between each test to ensure adequate recovery. The deceleration deficit is calculated as the difference between full approach time and 15 m linear sprint time.

### Isometric mid-thigh pull test

**FIGURE 3:**
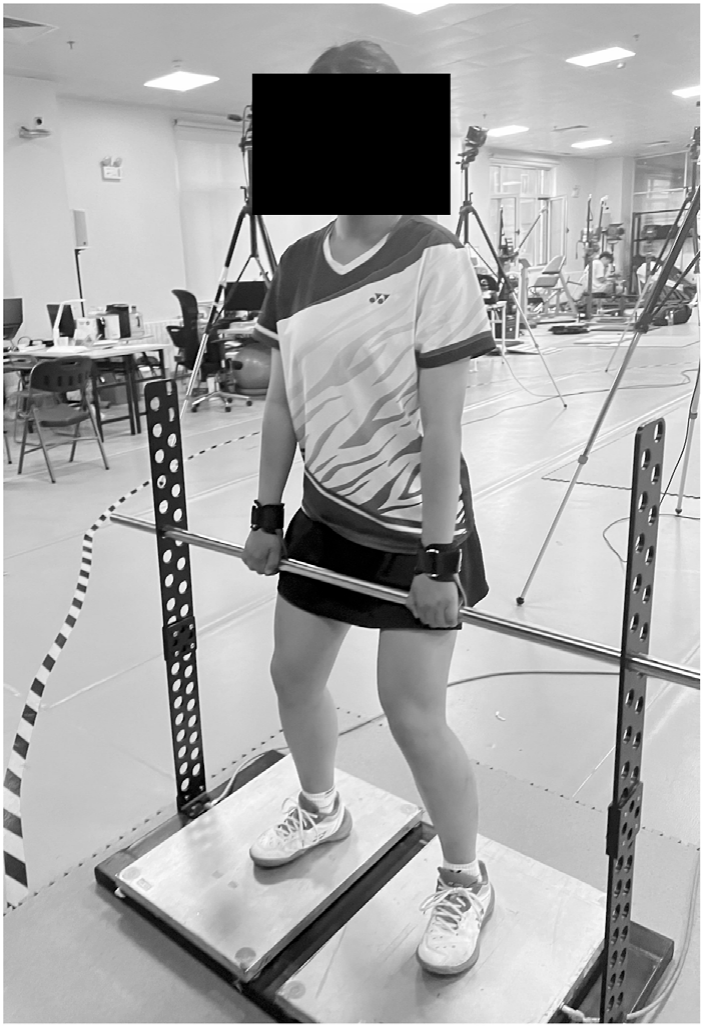
IMTP test.

The IMTP is a multi-joint exercise test used to assess whole-body strength and force production capabilities (Martin and Beckham, 2020). As shown in Figure 3, the IMTP testing equipment is fixed on a force plate (Kistler 9281CA, Switzerland, 1000 Hz). Before the test, the procedure is explained to players, and they are allowed to familiarize themselves with the process. During the test, players stand on the force plate with their knees at an angle of 130–140° and their torso upright, similar to the second pull phase of a clean lift. After assuming the correct starting position, researchers give the command to start, and players pull the bar with maximal effort for 3 s. Throughout the isometric mid-thigh pull, researchers provide continuous verbal encouragement to ensure players exert maximum effort. Each player performs three tests, with a 5-minute rest period between each test to ensure full recovery, and the average peak force of the best two performances was recorded and analyzed.

### Dynamic posture stability test

The test was used to assess the dynamic balance of players by measuring the DPSI (Sell, 2012). Participants stood on an in-ground force plate (Kistler 9281CA, Switzerland, 1000 Hz) and then jumped forward or laterally, landing on their dominant or non-dominant leg, and standing for 10 s. The distance between the jumping line and the center of the force platform was 40% of the participant’s height (cm) (Sell, 2012). A hurdle was placed at the midpoint between the jumping line and the force platform. The hurdle height was 30 cm for forward jumps and 15 cm for lateral jumps (Lu et al., 2022). All players were asked to complete each type of jump three times, and the average was taken for data analysis. The time-series data of ground reaction force (GRF) were collected within 10 s after the players landed on the force plate with a single leg.

Based on Wikstrom et al.’s method (Wikstrom et al., 2005), the DPSI was calculated from the GRF curve within 3 s after touchdown (the time when the GRF value exceeded 5% of body weight). Here, BW is the body weight, and GRF_x_, GRF_y_, and GRF_z_ are the GRF in anterior-posterior, medial-lateral, and vertical directions, respectively. The dynamic postural stability indexes of the forward jump (DF-DPSI) and lateral jump (DL-DPSI) were calculated.

$$DPSI=\frac{\sqrt{{\sum \left(0-GRF_{x}\right)}^{2}+{\sum \left(0-GRF_{y}\right)}^{2}+{\sum \left(0-GRF_{z}\right)}^{2}}}{number\;of\;data\;points}\times \frac{1}{body\;weight}$$

Generally, smaller DPSI displacement values indicate better stability and postural control upon single-leg landing.

### Statistical analysis

Experimental data were analysed using the IBM SPSS statistical software package (version 27.0, Chicago, IL, United States). All data were presented as means and standard deviations (SD). To examine the effects of two types of resistance training on deceleration and dynamic performance, a two-way repeated-measures ANOVA (group × time) was performed, with the Greenhouse-Geisser adjustment applied. The dependent variables for each model included DPSI (front and lateral, dominant and non-dominant leg), DD, time of the 5-0-5 COD test, and peak force of IMTP. The model effects included group, time, and their interaction. Partial *η* ^2^ was applied to estimate effect sizes (ES) for the time × group interaction effects, with thresholds interpreted as follows: <0.06 (small), <0.14 (moderate), and ≥0.14 (large) (Cohen, 1988). When a significant effect was detected, Bonferroni *post-hoc* correction was applied to identify pairwise differences. The absolute value of each test result was used to calculate the ES for both within- and between-group comparisons, expressed as Cohen’s d. Cohen’s d was interpreted as follows: <0.2 as trivial, 0.2–0.6 as small, 0.6–1.2 as moderate, 1.2–2.0 as large, and >2.0 as very large (Hopkins et al., 2009).

## Results

### Characteristics of study participants

All participants successfully completed the two exercise interventions. However, one player in the FT group was excluded because he did not compete the post-test due to illness. The physical characteristics of the participants who completed all tests are summarized in Table 1.

### 5-0-5 COD test

Significant effects of time (*p* < 0.001, ES = 1.62) were observed on the performance of 5-0-5 COD test, while no significant interaction between group and time was found (*p* = 0.646, partial *η* ^2^ = 0.01). Specifically, the *post hoc* analysis revealed that for both FT and RT group, 5-0-5 COD test were significantly greater than baseline (*p* < 0.001, ES = 1.53 vs *p* < 0.001, ES = 1.61), and there was no significant difference between the FT and RT groups after the intervention (*p* = 0.374, ES = 0.45) (Table 3).

**TABLE 3:** The assessment results for FT group and RT group before and after the 6-week training.

	FT (n = 8)		RT (n = 9)				Time		Group*Time	
	Pre	Post	Cohen’ d	Pre	Post	Cohen’ d	*p*	Cohen’ d	*p*	Cohen’ d
IMTP (n)	2,191.42 ± 527.98	2,351.87 ± 525.71^*^	0.475	2,135.05 ± 334.58	2,103.93 ± 400.27	0.042	0.049	0.248	0.007	0.420
5–0-5 COD test (s)	2.63 ± 0.13	2.43 ± 0.13^*^	0.589	2.59 ± 0.11	2.36 ± 0.17^*^	0.676	<0.001	0.776	0.646	0.014
Deceleration Deficit	0.61 ± 0.11	0.45 ± 0.20^*^	0.251	0.67 ± 0.21	0.57 ± 0.17	0.110	0.027	0.302	0.559	0.025
DF-DPSI	1.08 ± 0.16	0.76 ± 0.17^*#^	0.916	1.06 ± 0.16	1.00 ± 0.15^*^	0.297	<0.001	0.890	<0.001	0.792
DL-DPSI	1.18 ± 0.12	0.95 ± 0.11^*#^	0.796	1.17 ± 0.07	1.08 ± 0.13^*^	0.345	<0.001	0.789	0.002	0.469
NF-DPSI	1.15 ± 0.18	0.89 ± 0.12^*#^	0.634	1.14 ± 0.16	1.20 ± 0.12	0.097	0.012	0.349	<0.001	0.583
NL-DPSI	1.22 ± 0.16	0.94 ± 0.10^*#^	0.672	1.10 ± 0.14	1.13 ± 0.14	0.021	0.002	0.470	<0.001	0.566

Note: * Statistically significant difference between pre- and post-test, *p* < 0.05. # Statistically significant difference between FT, group and RT, group, *p* < 0.05. D, dominant foot; N, non-dominant foot; F, forward jump; L, lateral jump; COP, center of pressure.

### Deceleration deficit

Significant effects of time (*p* = 0.027, ES = 0.72) were observed on the performance of deceleration time, while no significant interaction between group and time was found (*p* = 0.559, partial *η* ^2^ = 0.03). Specifically, the deceleration deficit of FT group showed significant difference compared to baseline (*p* = 0.048, ES = 0.99), while no significant effects were observed for the RT group after the intervention (*p* = 0.209, ES = 0.52). There was no significant difference between the FT and RT groups after the intervention (*p* = 0.220, ES = 0.64).

### Peak force of IMTP

Significant effects of time (*p* = 0.049, ES = 0.12) were observed on the peak force of IMTP, and a significant interaction between group and time (*p* = 0.007, partial *η* ^2^ = 0.42) was also observed. Specifically, *post hoc* analysis revealed that for FT group, IMTP were significantly greater than baseline (*p* = 0.003, ES = 0.30), while no significant effects were observed for the RT group after the intervention (*p* = 0.447, ES = 0.08). There was no significant difference between the FT and RT groups after the intervention (*p* = 0.301, ES = 0.53).

### Dynamic posture stability index

The results of ANOVA models on dominant leg jumps, both forward and lateral, showed the significant effects of time (DF-DPSI: *p* < 0.001, ES = 0.99; DL-DPSI: *p* < 0.001, ES = 1.23). A significant interaction between group and time (DF-DPSI: p < 0.001, partial *η* ^2^ = 0.79; DL-DPSI: *p* = 0.002, partial *η* ^2^ = 0.47) was also observed (Table 3). The *post hoc* analysis revealed that the participants who received FT had significantly lower DF-DPSI and DL-DPSI compared to their baseline performance (DF-DPSI: *p* < 0.001, ES = 1.94; DL-DPSI: *p* < 0.001, ES = 2.00) and those who received RT (DF-DPSI: *p* = 0.007, ES = 1.50; DL-DPSI: *p* = 0.034, ES = 1.14).

The results of ANOVA models on non-dominant leg jumps, both forward and lateral, showed the significant effects of time (NF-DPSI: *p* = 0.012, ES = 0.48; NL-DPSI: *p* = 0.002, ES = 0.75). A significant interaction between group and time (NF-DPSI: *p* < 0.001, partial *η* ^2^ = 0.58; NL-DPSI: *p* < 0.001, partial *η* ^2^ = 0.57) was also observed (Table 3). The *post hoc* analysis revealed that the participants who received FT had significantly lower NF-DPSI and NL-DPSI compared to their baseline performance (NF-DPSI: *p* < 0.001, ES = 1.70; NL-DPSI: *p* < 0.001, ES = 2.10) and those who received RT (NF-DPSI: *p* < 0.001, ES = 2.53; NL-DPSI: *p* = 0.007, ES = 2.53).

## Discussion

The purpose of the study was to investigate the effectiveness of a 6-week flywheel resistance training program on the deceleration ability and dynamic balance performance of elite badminton players. Based on the results, we found that FRT is more effective in improving deceleration ability, peak force of IMTP, and dynamic balance of players when compared to TRT. This suggests that FRT is a promising strategy to enhance the functionality of elite badminton players and potentially improve their on-court performance.

By comparing the delta% before and after training of the FT and RT (Table 4), we can find that compared to TRT, a 6-week flywheel resistance training program significantly improved deceleration performance in elite badminton athletes. Compared to the 5-0-5 COD test, the Deceleration Deficit provides a more direct assessment of changes in deceleration and braking abilities results from training, as it addresses the limitation of using total time as the outcome variable in traditional COD tests (Clarke et al., 2022). The results revealed a significant increase in 5-0-5 COD performance in both the FT group and RT group, with only the FT group showing a larger improvement in DD. This aligns with previous research, as Tous-Fajardo et al. (2016) suggested that combining flywheel resistance training with vibration training can serve as a feasible supplementary method to enhance specific soccer performance metrics, such as speed and deceleration abilities. de Hoyo et al. (2015) demonstrated that a 10-week eccentric overload flywheel training program can significantly improve jumping ability and linear-sprinting speed in adolescent soccer players. In a badminton match, players need to quickly judge the direction and trajectory of the ball before hitting it, then perform rapid starts, sprints, and frequent deceleration and braking processes in a short period on the court (Cabello Manrique and González-Badillo, 2003; Lu et al., 2022). These movements create opportunities for players to transition to the next high-intensity action while executing technical and tactical maneuvers (Ade et al., 2016), thereby enhancing their defensive and offensive capabilities. In summary, this study explores the efficacy of FRT on deceleration abilities through the DD test, offering a novel approach for future research in the field of deceleration and braking, and highlights the significant importance of FRT in improving the athletic performance of badminton players.

**TABLE 4:** Delta% change performance for FT group and RT group.

	FT (n = 8)	RT (n = 9)
IMTP (n)	7.32%	−1.46%
5-0-5 COD test (s)	7.61%	8.80%
Deceleration Deficit	9.12%	6.61%
DF-DPSI	29.82%	5.66%
DL-DPSI	20.10%	7.10%
NF-DPSI	21.94%	5.16%
NL-DPSI	23.03%	2.46%

Dynamic balance is another important factor for outstanding performance in badminton competitions (Lu et al., 2022). The results further revealed a significant increase in DPSI performance for the both dominant and non-dominant legs in the FT group, while the RT group showed meaningful adaptation only in the dominant leg. It can be inferred from this result that badminton-specific dynamic balance performance improved after TRT, but not as much as with a FRT protocol. This is consistent with previous research where significant adaptations were reported (Kaux et al., 2013; Martinez-Aranda and Fernandez-Gonzalo, 2017). The performance of DPSI reflects the ability of players to maintain landing stability and balance (Pau et al., 2019; Wikstrom et al., 2005). Studies have identified the four major neuromuscular performance determinants of deceleration: eccentric strength, reactive strength, power, and dynamic balance (Harper et al., 2022). TRT often focuses only on musculoskeletal strength, while FRT can improve neuromuscular strength and coordination, enhancing the expression of tendon and muscle movement capabilities (Beato et al., 2024), and simultaneously target multiple aspects of dynamic balance. This can thus benefit the capacity of the postural control system. For example, Fernandez-Gonzalo et al. (Fernandez-Gonzalo et al., 2016) observed that FRT is a powerful aid in regaining muscle mass, function, and functional performance in individuals with stroke. Studies have shown that high-risk landing actions or inadequate neuromuscular adaptation during exercise can lead to greater stress on the ACL of the knee (Harper et al., 2019). Therefore, increases in muscle strength and improvements in neuromuscular recruitment can better maintain knee stability, which is crucial for preventing lower limb injuries (Fort-Vanmeerhaeghe et al., 2016). In this study, TRT did not effectively increase peak force of IMTP, possibly because the elite badminton athletes selected had already undergone years of training, making further improvements in peak force less significant. However, FRT, with its eccentric overload, effectively enhanced the peak force of these elite athletes. Carroll et al. (2019) found that during the eccentric overload phase of FRT, the eccentric load of each cycle is approximately 1.25 times the concentric load, and players must also overcome the adaptive resistance generated by the flywheel’s cyclic motion. As previously mentioned, key factors in improving horizontal deceleration ability include the control and reduction of braking force. Increasing peak force can enhance the ability of elite badminton athletes to pre-activate and pre-stretch muscles before foot contact, thereby improving braking force control and deceleration ability during movement. Incorporating FRT into athletes’ training programs has shown improvements in muscle strength, particularly during the eccentric phase of muscle contraction, which is crucial for effective deceleration and energy absorption during competitions. In this regard, the flywheel resistance training paradigm could be more preferable for the complex, high-speed, unilateral, repetitive dynamic badminton movements that request a high extent of postural control.

The results of our study evidenced the promising effects of the flywheel resistance training paradigm on badminton deceleration and dynamic balance performance, suggesting that it is feasible to apply FRT into existed strength training routines of athletes. Fitness coaches are advised to vary the format and sequence of exercises, as well as the training volumes and intensities, to introduce more stimuli. Despite the new information about the effect of flywheel resistance training on badminton deceleration and dynamic balance, several limitations need to be acknowledged. First, because most elite badminton athletes usually have fixed match and training schedules (traveling to compete and train) that cannot be alternated, the study could only include a comparatively small sample size. Second, it should be noted that an experiment period of 6 weeks was chosen to verify that whether a shorter intervention than the commonly-used 8 weeks could induce favorable adaptation, as the experiment schedule had to be acceptable for both the coaches and athletes. Researchers should, therefore, be cautious when interpreting and generalizing the current findings.

## Conclusion

This pilot study demonstrates that, compared to traditional resistance training, flywheel resistance training enhances deceleration performance and improves dynamic balance in elite badminton athletes. These findings offer valuable insights that can inform the design of future, larger-scale studies aimed at further validating the effects of flywheel resistance training on athletic performance.

## Data Availability

The original contributions presented in the study are included in the article/supplementary material, further inquiries can be directed to the corresponding author.
